# Supplementary figures and images for: A genetic modifier links integrin α5 to the phenotypic variation in fibronectin 1a mutant zebrafish
Source: PLoS Genet. 2025 Jun 23;21(6):e1011747. doi: 10.1371/journal.pgen.1011747 (PMC12212883; doi:10.1371/journal.pgen.1011747)

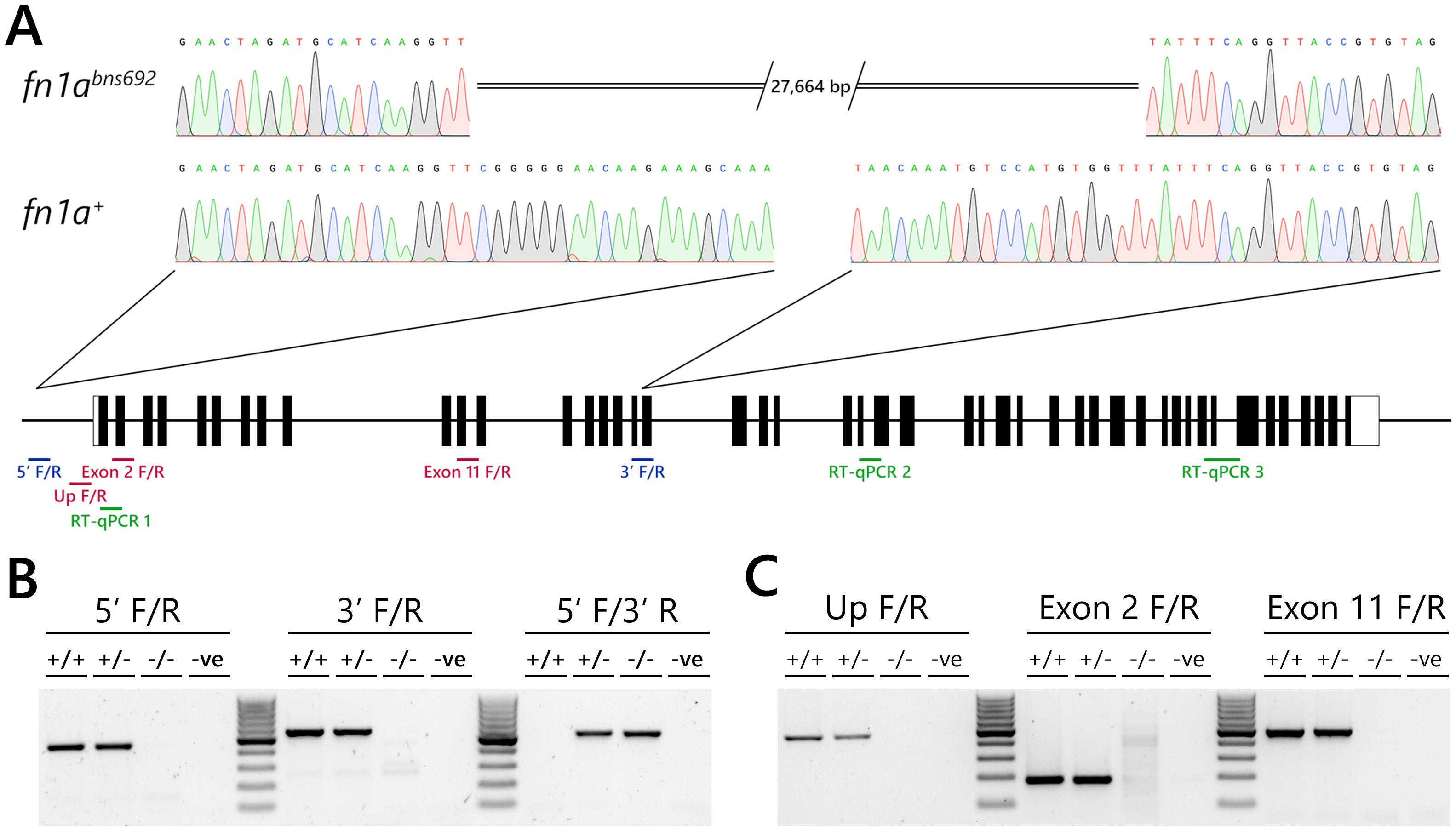

Supplement: S1 Fig — (A) Schematic showing the fn1abns692 27,664 bp deletion. Exons (n = 46) are indicated by black boxes. The 5’ and 3’ UTRs are indicated by open boxes. The position of the deletion as well as of the PCR primers used to genotype fn1abns692 mutants (blue), to test for the possible reintegration of the deleted region (red), and for the RT-qPCR analysis (green) is indicated. (B) Genotyping of fn1abns692 homozygous WT, heterozygous, and mutant embryos by PCR. (C) Three primer pairs were used to test for the possible reintegration of the deleted region by PCR. GeneRuler 100 bp DNA ladder is present in all gels. (TIF) [file pgen.1011747.s001.tif]

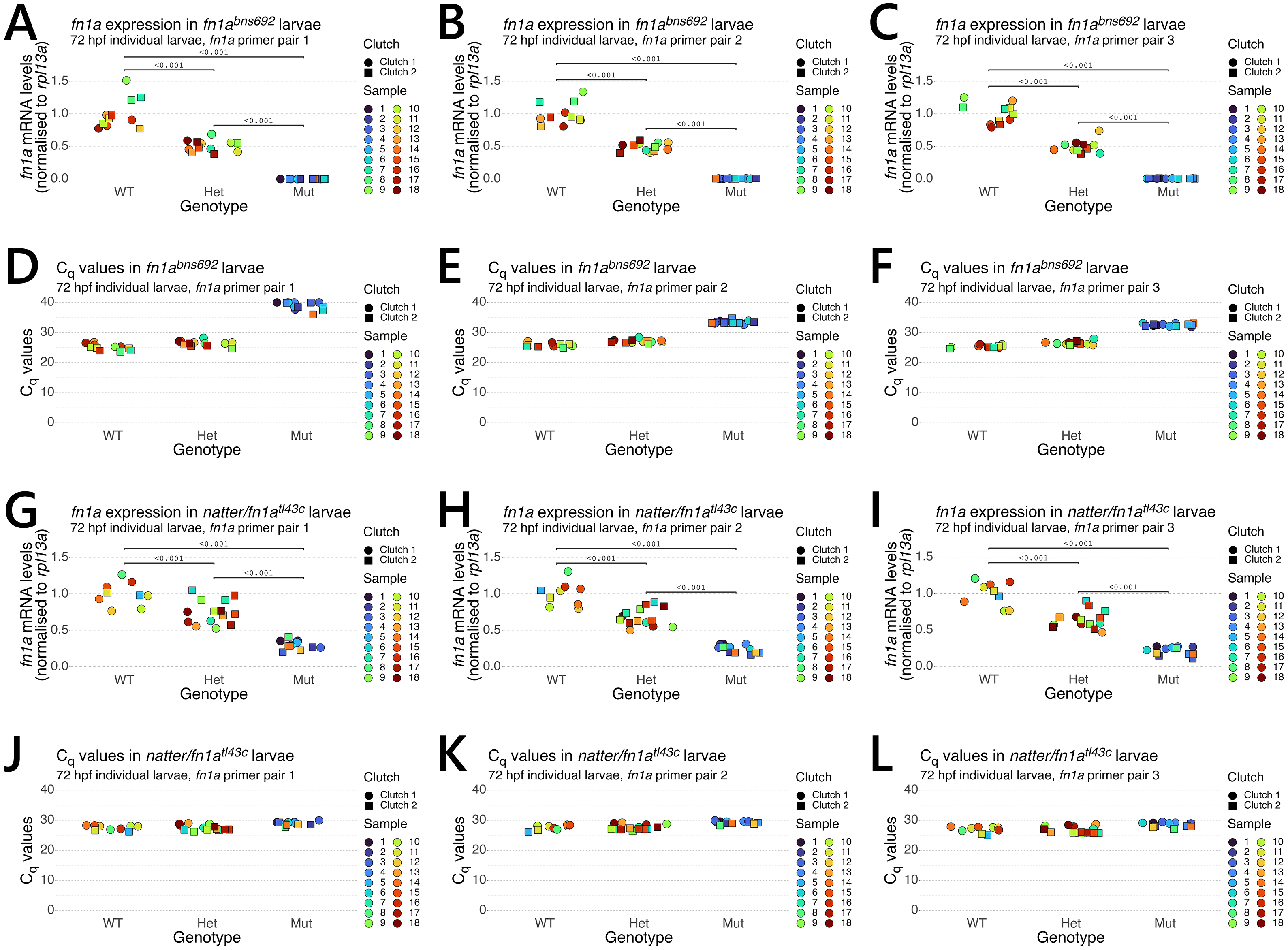

Supplement: S2 Fig — fn1a mRNA levels were examined in 72 hpf larvae with three sets of RT-qPCR primers (see annotation in S1 Fig). Each data point represents an individual larva. Expression is relative to homozygous WT siblings in each clutch. Two clutches were collected from single pair fn1abns692 heterozygous intercrosses in (A-F), and single pair natter/fn1atl43c heterozygous intercrosses in (G-L). All primer pairs reveal an almost complete loss of fn1a expression in fn1abns692 mutants (A-C) when compared with WT siblings and with natter/fn1atl43c mutants (G-I). However, the Cq values indicate that low mRNA levels are detectable from the 3’ fn1a sequence in fn1abns692 mutants – compare (D) with (E) and (F), whereas Cq values are consistent for all three primer pairs used in natter/fn1atl43c mutants (J-L). All p-values were calculated using one-way ANOVA with Tukey’s post-hoc test for multiple comparisons. (TIF) [file pgen.1011747.s002.tif]

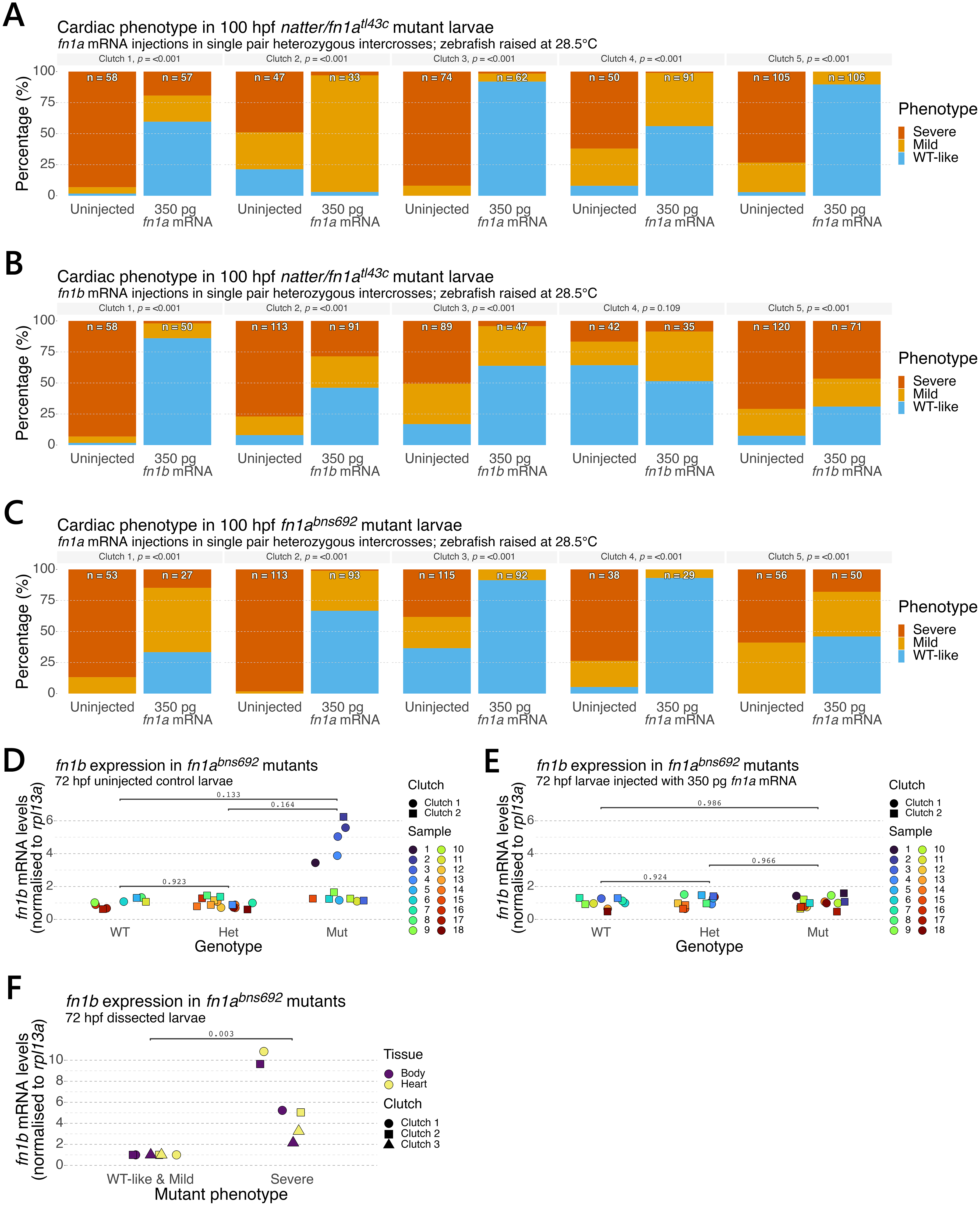

Supplement: S3 Fig — fn1a and fn1b mRNA injections rescue the fn1a mutant cardiac phenotype. Quantification of the 100 hpf cardiac phenotype in uninjected and fn1a (A), or fn1b (B) mRNA injected mutant larvae from five single pair natter/fn1atl43c mutant incrosses. (C) Quantification of the 100 hpf cardiac phenotype in uninjected and fn1a mRNA injected mutant larvae from five single pair fn1abns692 mutant incrosses. The numbers on each bar indicate the total number of larvae assessed. fn1b mRNA levels were analysed by RT-qPCR in uninjected (D) and 350 pg fn1a mRNA injected (E) 72 hpf fn1abns692 mutant larvae and WT siblings collected from two single pair heterozygous intercrosses. Two clutches were collected and analysed. Each data point represents an individual larva. Expression is relative to homozygous WT siblings in each clutch. (F) fn1b mRNA levels were analysed by RT-qPCR in the hearts and bodies of dissected 72 hpf fn1abns692 mutant larvae collected from single pair mutant incrosses. Each data point represents pooled tissue; 5–10 larvae were dissected for each pool. Mutants displaying the WT-like and mild phenotypes were pooled together. Severe mutants were pooled separately. Expression is relative to the WT-like/mild mutant pool in each clutch. For (A-C), p-values were calculated using a chi-squared test; for (D-E), p-values were calculated by one-way ANOVA with Tukey’s post-hoc test for multiple comparisons; and for (F), p-values were calculated by a Wilcoxon rank-sum test. (TIF) [file pgen.1011747.s003.tif]

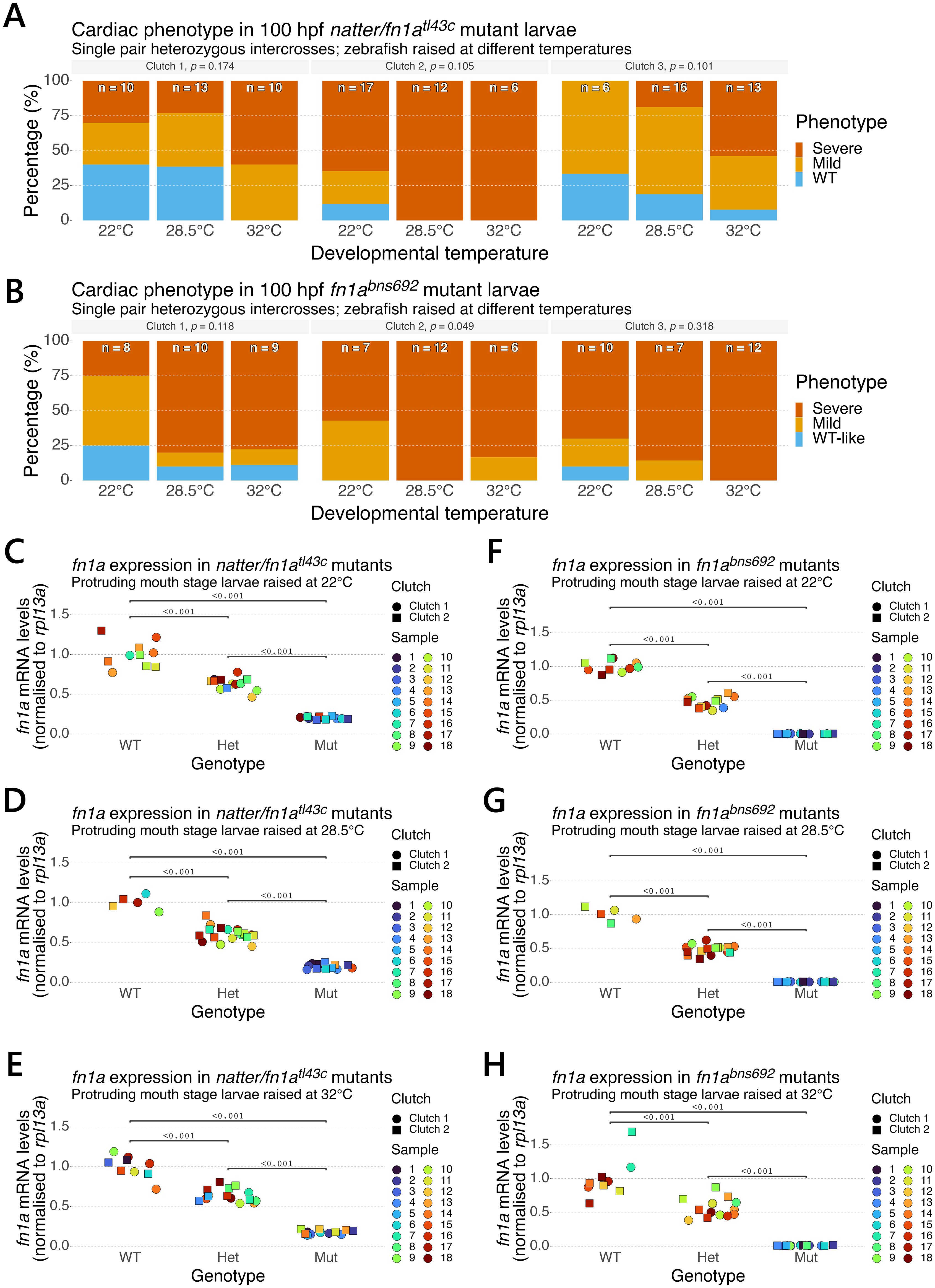

Supplement: S4 Fig — Quantification of the 100 hpf cardiac phenotype in natter/fn1atl43c (A) and fn1abns692 (B) mutants from three single pair heterozygous intercrosses. Each clutch was split into three dishes at an early blastula stage and raised at 28.5°C until the embryos reached the bud stage (10 hpf). Dishes were then incubated at different temperatures until the larvae reached the equivalent of a 100 hpf larva raised at 28.5°C, and were then phenotyped. The numbers on each bar indicate the total number of mutant larvae assessed. fn1a mRNA levels were examined at the protruding mouth stage (72 hpf when raised at 28.5°C) in two clutches from single pair natter/fn1atl43c (C-E) and fn1abns692 (F-H) heterozygous intercrosses. Each clutch was split into three dishes at an early blastula stage and raised at 28.5°C until they reached the bud stage (10 hpf). Dishes were then incubated at 22°C (C, F), 28.5°C (D, G), or 32°C (E, H) until they reached the protruding mouth stage, and were then collected for RT-qPCR analysis. Each data point represents an individual larva. Expression is relative to homozygous WT siblings in each clutch. p-values were calculated using a chi-squared test in (A) and (B), and one-way ANOVA with Tukey’s post-hoc test for multiple comparisons for (C-H). (TIF) [file pgen.1011747.s004.tif]

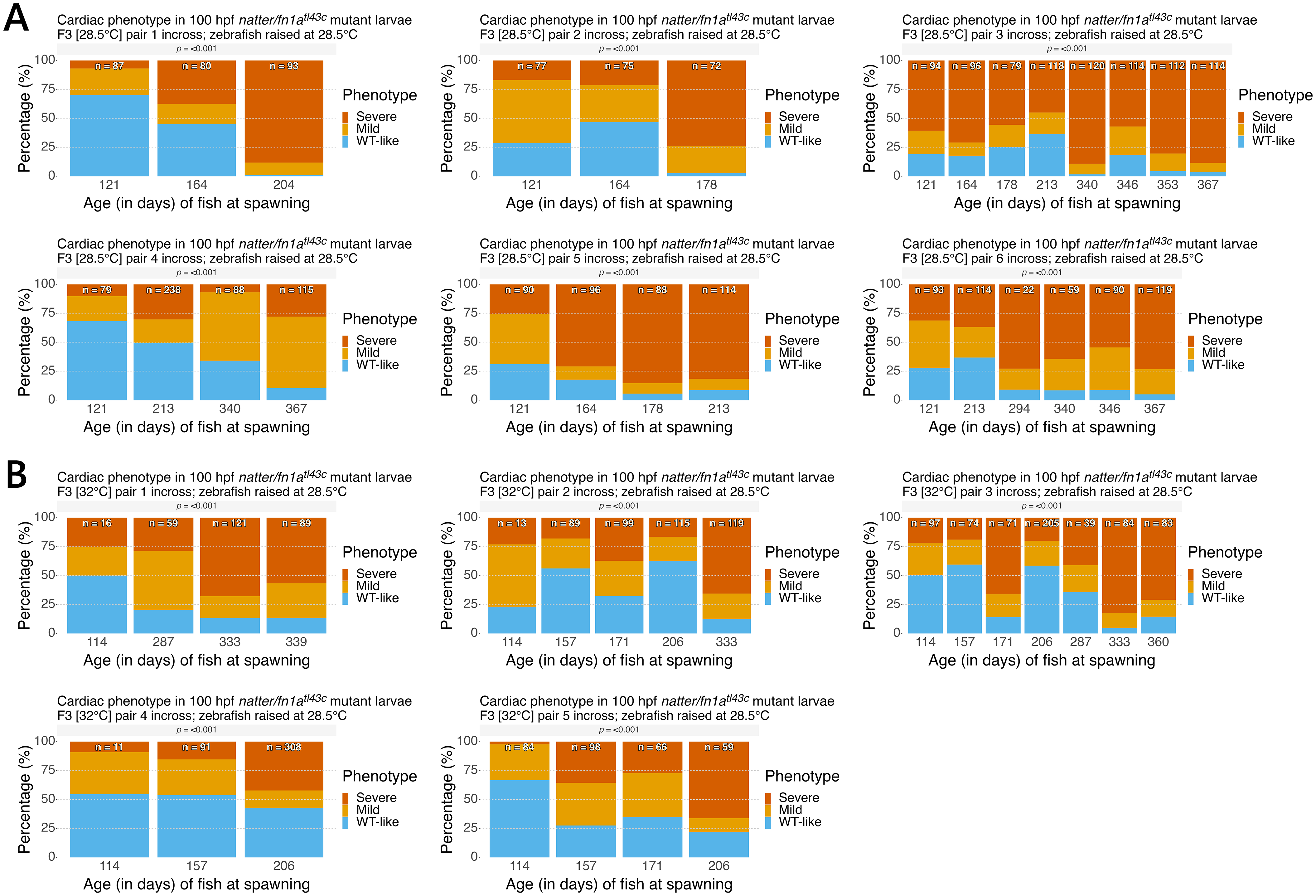

Supplement: S5 Fig — Single pair F3 [28.5°C] (A) and F3 [32°C] (B) natter/fn1atl43c mutants were repeatedly incrossed over multiple weeks and the resulting larvae phenotyped at 100 hpf. The numbers on each bar indicate the total number of larvae assessed. All p-values were calculated using a chi-squared test. (TIF) [file pgen.1011747.s005.tif]

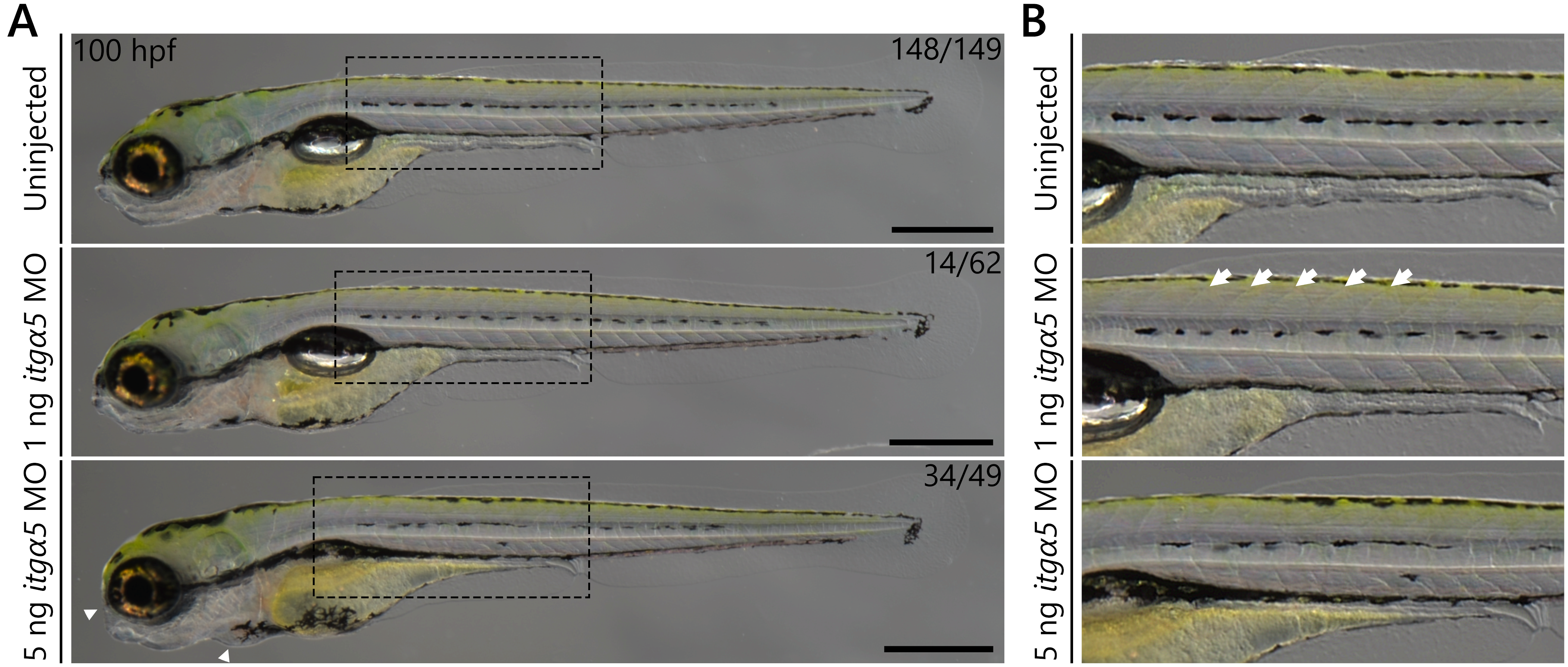

Supplement: S6 Fig — (A) Images of 100 hpf uninjected, 1 ng itgα5 MO injected, and 5 ng itgα5 MO injected larvae from single pair WT incrosses are shown. Outlined regions in (A) are enlarged in (B) to show the somite phenotype. While the majority of 1 ng itgα5 MO injected larvae resembled the uninjected control, a small proportion developed U-shaped somite boundaries (white arrows in (B)) as pictured. 5 ng itgα5 MO injected larvae failed to inflate their swim bladder, developed pericardial oedema and craniofacial defects (white arrowheads in (A)), and displayed defects in somitogenesis. Lateral views are shown with anterior to the left. The proportion of larvae matching the image shown is indicated in the top right corner of each image. Scale bars, 1 mm. (TIF) [file pgen.1011747.s006.tif]
