# Supplementary material for: A genetic modifier links integrin α5 to the phenotypic variation in fibronectin 1a mutant zebrafish
Source: PLoS Genet. 2025 Jun 23;21(6):e1011747. doi: 10.1371/journal.pgen.1011747 (PMC12212883; doi:10.1371/journal.pgen.1011747)
Supplement: Attachment — Submitted filename: 2025-05-20_revised_cover_letter.pdf [file pgen.1011747.s013.pdf]

MAX PLANCK INSTITUTE
FOR HEART AND LUNG RESEARCH

Max Planck Institute for Heart and Lung Research
Ludwigstrasse 43 · D-61231 Bad Nauheim

Aimée Dudley
Anne Goriely
Editors-in-Chief
Ophir Klein, Academic Editor
Fengwei Yu, Section Editor
PLOS Genetics

20 May 2025

Dear Dr. Dudley, dear Dr. Goriely, dear Ophir, dear Dr. Yu,

Please find attached our revised manuscript entitled "**A genetic modifier links *integrin α5* to the phenotypic variation in *fibronectin 1a* mutant zebrafish**" for consideration as a research article in *PLOS Genetics*. We thank you for your initial positive response, which has given us the opportunity to improve our manuscript.

We also want to thank the reviewers for their positive remarks as well as their helpful suggestions and advice. Please find below our point-by-point response to the reviewers' comments.

We have now addressed all of the major and minor comments from the reviewers. In particular, we have analysed *integrin α5* (*itgα5*) mRNA expression in *natter/fibronectin 1a* (*fn1a*) mutants sorted by phenotype, and overexpressed *itga5* mRNA in *natter/fn1a* mutants. In addition, we have clarified a number of important points and added references as suggested by the reviewers. These new data strengthen the major finding of our manuscript, namely, the identification of a genetic modifier of the *natter/fn1a* mutant that maps to the *itgα5* locus.

All major changes in the main text are highlighted in yellow.

Thank you for considering our revised manuscript.

Sincerely,

Samuel Capon and Didier Stainier

Max Planck Institute for Heart and Lung Research
Prof. Didier Stainier, Ph.D. | Director | Department of Developmental Genetics
Ludwigstrasse 43 | D-61231 Bad Nauheim
didier.stainier@mpi-bn.mpg.de | Tel: +49(0)6032 705-1302 | Fax: +49 (0)6032 705-1304 | www.mpi-hlr.de
